# The Impact of COVID-19 on Primary Care General Practice Consultations in a Teaching Hospital in Shanghai, China

**DOI:** 10.3389/fmed.2021.642496

**Published:** 2021-03-26

**Authors:** Zhongqing Xu, Jingchun Fan, Jingjing Ding, Xianzhen Feng, Shunyu Tao, Jun Zhou, Lingmei Qian, Kun Tao, Brett D. Hambly, Shisan Bao

**Affiliations:** ^1^Department of General Practice, Tongren Hospital, Shanghai, China; ^2^Discipline of General Practice, School of Medicine, Shanghai Jiao Tong University, Shanghai, China; ^3^Center for Community Health Care, China Hospital Development Institute, Shanghai Jiao Tong University, Shanghai, China; ^4^School of Public Health, Gansu University of Chinese Medicine, Lanzhou, China; ^5^Center for Evidence-Based Medicine, Gansu University of Chinese Medicine, Lanzhou, China; ^6^School of Public Health, Chongqing Medical University, Chongqing, China; ^7^Discipline of Pathology, Charles Perkin Centre, D17, Faculty of Medicine and Health, School of Medical Sciences, The University of Sydney, Darlington, NSW, Australia

**Keywords:** COVID-19, GP, primary care, lockdown, demographic pattern, psychological disorders

## Abstract

**Background:** The COVID-19 (2019 novel coronavirus disease) pandemic is deeply concerning because of its massive mortality and morbidity, creating adverse perceptions among patients likely to impact on their overall medical care. Thus, we evaluated the impact of the COVID-19 pandemic on the pattern of primary care consultations within a Shanghai health district.

**Methods:** A retrospective observational cohort study was performed, with data analyzed concerning the pattern of patient visits to general practitioners within the Tongren Hospital network (the sole provider of general practice to the population of 700,000). Data from all general practice consultations for adults were collected for the first 6 months of 2020, which included a 60-day lockdown period (January 24–March 24, 2020) and compared to corresponding data from the first 6 months of 2019. We evaluated changes to the numbers and patterns of primary care consultations, including subgroup analysis based on age, sex, and primary diagnosis.

**Results:** A substantial reduction in patient visits, associated with increased median age, was observed during the first wave of the pandemic in the first 6 months of 2020, compared to the same interval during 2019. Additionally, reduced reappointments and waiting times, but increased costs per visit were observed. When analyzed by primary disease diagnosis, patient visits were reduced for all the major systems. The most striking visit reductions were in cardiovascular, respiratory, endocrine, and gastrointestinal diseases. However, psychological disorders were increased following lockdown, but there was also a dramatic fall in consultations for depression. Reduced monthly patient numbers correlated with both rate of reappointment and average waiting time during the first 6 months of both 2019 and 2020, but an inverse correlation was observed between cost per visit and monthly patient numbers. Specifically during the lockdown period, there was ~50% reduced patient visits.

**Conclusions:** The lockdown has had a serious impact on patients' physical and psychological health. Our analysis provides objective health-related data that may inform the current controversy concerning the balance between the detrimental effects of the use of lockdown vs. the use of a more targeted approach to eliminate viral transmission. These data may improve decision-making in medical practice, policy, and education.

## Background

The 2019 novel coronavirus disease (COVID-19), caused by infection with the severe acute respiratory syndrome coronavirus 2 (SARS-CoV-2) virus, was originally discovered in late December 2019 (1). Because of a lack of sufficient knowledge of the SARS-CoV-2 virus transmission dynamics during the initial stages, the outbreak of COVID-19 was transmitted at extraordinary speed, i.e., reaching every province/autonomous region in China within months (2). Currently (as at January 21, 2021), data on the extent of the pandemic are as follows: the pandemic has involved 215 countries and territories, with a reported total of 95,321,880 confirmed cases; including a total of 2,058,227 deaths (3).

Shanghai, located in the eastern coastal region of China, is one of the largest modern cities in China with a population of ~25 million (including regular and transient residents) (4). Shanghai contributes to ~4% of the GDP of China (5). The first officially confirmed COVID-19 case was reported in Shanghai on January 20, 2020 (6). In an attempt to stop viral transmission, the highest level of public health emergency response was initiated by the Shanghai Government, implementing a strict lockdown that commenced on January 24, 2020, including the mandatory wearing of face masks in public, no public gatherings, maintaining social distancing, and school and factory closures (7, 8). Only absolutely necessary grocery shops were allowed to open (9). Because of the effectiveness of the initial strict lockdown measures, there have subsequently been almost no new indigenous COVID-19 cases reported in Shanghai, with only a few cases of COVID-19 imported from other cities/regions (10). The total number of COVID-19 cases confirmed in Shanghai was 394 or 712 on March 21 or June 30, 2020, respectively (11, 12). Thus, the control measures that have been implemented have been extremely successful, particularly considering the size of the whole population of Shanghai, of ~25 million. As a result of this success, the public health emergency response was downgraded to class 2 and then class 3 on March 24 and May 9, 2020 (10, 13, 14), respectively, and almost all schools in Shanghai have been allowed to reopen, following the initial use of online teaching only during March and April 2020 (15).

Transmission of the highly infectious SARS-CoV-2 virus is mainly due to its S spiking protein, which enables binding to ACE2 on host cells (16). This mechanism of transmission is consistent with the most common clinical presentations of COVID-19 being in the respiratory, cardiovascular, and/or renal systems (17), where ACE2 is highly expressed, especially among those patients who have preexisting chronic conditions within these systems.

Worldwide the key control strategy for the pandemic has been the implementation of extensive national or regional lockdowns and home confinements, in an attempt to control further disease transmission, which has been implemented in almost every country/region where an outbreak of COVID-19 has occurred (18). Thus, the pandemic of COVID-19 has become the most challenging and frightening event of this millennium to almost all people throughout the world, resulting in unprecedented global anxiety and distress within communities worldwide, which represents a natural psychological response to what the population believes are randomly changing conditions over which individuals have almost no control (19). There are two major contributing factors to the abnormal psychosomatic outcomes within the general population, which are expected to continue to increase significantly, these being the continuing evolution of the COVID-19 pandemic itself, as well as the extremely stressful information concerning COVID-19, which continues to bombard the population through the media (20). Thus, there is a special term for this psychological disorder, i.e., coronaphobia (21).

However, scientific and public health experts have questioned the harsh lockdown approach to the control of the pandemic, most recently in the Great Barrington Declaration, authored by Kulldorff, Gupta, and Bhattacharya, and released on October 4, 2020. The declaration is critical of the damaging physical and mental health impacts of the prevailing COVID-19 policies that incorporate a strict lockdown, but rather recommends the use of focused protection for at-risk groups only (22). Qualified support has been provided by the World Health Organization (WHO), who have called for world leaders to stop locking down their countries and economies. Dr. Nabarro, the Senior UN WHO System Coordinator for Avian and Pandemic Influenza, has indicated that the lockdown has not achieved the intended levels of lifesaving, but has dramatically and potentially catastrophically increased extreme poverty (23). An additional source of considerable concern and consequent anxiety has been scientific uncertainty concerning the path to effective definitive pandemic control; for example, a limited number of reports of reinfection with SARS-CoV-2 has been placing into doubt the ability of vaccination and consequent herd immunity to quickly and effectively control the pandemic and provide a further challenge to the current rationale for continued lockdowns (24).

Within Shanghai, the traditional and most effective way for both primary care and specialist medical consultation and treatment to occur has been via visits in person to hospital outpatient departments. However, during the pandemic, alternative approaches have been introduced and encouraged for the conduct of safer medical practice, for example, telephone and/or online consultations (25). A combination of psychological and physical issues associated with the pandemic may modify the choice of how patients may choose to consult with doctors and other health professionals. Thus, it remains to be clarified if COVID-19 has affected the demographics of patient visits to primary care general practice outpatient departments. Furthermore, the possibility that changes in the pattern of visits will modify the progression of diseases individually and across communities needs to be studied.

Despite the major achievement in controlling transmission of infection in Shanghai during the outbreak of COVID-19, with only 712 infections among a population of 25 million during the first wave, there were still substantial impacts of the pandemic on the general population, including patients' visits to hospitals. Shanghai Tongren Hospital, a general teaching hospital, is located in the Changning District, in the western region of Shanghai. The Department of General Practice within the hospital is the center for primary health care for the district and includes a network of 10 community health service centers (general practice outpatient departments), which manage the primary health care for all of the >700,000 people within the general population of this district. There are no private doctors or clinics within China, except for a few clinics run especially for overseas tourists visiting China. Thus, we are able to capture all of the general population primary care activity within the system. There are no obstacles to accessing the primary health system, as no appointment is needed prior to visiting general practitioners (GPs) in these hospital centers, although an online booking system is available. Access is at reasonable and affordable cost, subsidized by the government. Thus, the universality of the primary care system in China contrasts with that in many Western countries, where both cost and eligibility for services, often from multiple service providers, are a frequent characteristic of primary care. If the presenting medical issue was unable to be solved within the primary care environment, these patients were referred to specialists.

It is still unclear what the exact impact of the COVID-19 lockdown has been on the number of patient's visits, the pattern of visits, the pattern of different disease presentations, and the cost of patient visits, compared to the same period in the previous year. This study aimed to determine whether there were any changes in these parameters for outpatient visits to the primary health care centers during the pandemic, in comparison to data acquired during the same period in the previous year, prior to the COVID-19 outbreak in Shanghai. Because of the relatively large size of the population covered by the outpatient GP clinics network within Tongren Hospital, which is the only primary care service that covers almost 5% of the Shanghai population, the data are expected to provide some solid and convincing evidence to demonstrate the impact of COVID-19 within the general population, as well as provide supporting data for defining and updating recommendations for patient care for specific disease commodity packages, as defined by WHO. The Series of disease specific datasheets (DCPs) are a series of disease specific datasheets that list the critical commodities and the technical specifications recommended for each form of disease (26, 27).

Our data may provide some useful information for GPs to deal more effectively with COVID-19 and maintain a high level of health within the population, by adopting and adapting to different approaches compared to those used before.

## Methods

### Study Design

A retrospective simple cross-sectional observational cohort study was conducted within the Department of General Practice in Tongren Hospital in Shanghai and was approved by the Medical Ethics Committee of the hospital (no. 2020-079-01), strictly following the World Medical Association's Declaration of Helsinki. The information about the patients was obtained from the Center of Electronic Health Records in Tongren Hospital. All data obtained from patients were deidentified prior to analysis and strictly followed the guidelines of Helsinki. Written consent was obtained from all of the patients when they were registered with the clinics that the deidentified data can be used for public health purposes.

Data that were collected were from the Center database and included all the patient data for patients who visited GP outpatient clinics in person from January to June 2020, as well as the same period in 2019. As the pediatric outpatient department is a separate entity, the ages of the patients included in the current Outpatients study were all ≥12 years.

The inclusion criteria were that any of the patients who visited in the outpatient Department of General Practice for any medical problem were included within the period specified in the current study. There were no exclusions, except that all patients younger than 12 years were transferred to the pediatric department and allowed to utilize the adult Department of General Practice. Notably, we included all the outpatients who were transferred to being inpatients of the Department of General Practice in the current study, although the proportion of these patients was very small (<0.1%).

The cost per patient visit was obtained from the electronic health record system, including consultation fee, any laboratory tests, medication fee, and any related cost(s). The Chinese medical system is different from the Western system in that the prescribed medications are obtained in the pharmacy of the hospital. In addition, the patients with a medical insurance card from the government pay a relatively small amount of fees incurred above.

### Statistical Analysis

Quantitative variables were expressed as means with SEM, and qualitative variables were expressed as percentages. Differences were evaluated using the χ^2^-test for categorical variables and *t*-test for normally distributed variables. Correlations between two variables were detected using the Pearson test. For all the statistical analyses, *p* < 0.05 was considered significant. All analyses were performed using GraphPad Prism 8.0.1.

## Results

### Overall Outpatients Visits

**Figure 1:**
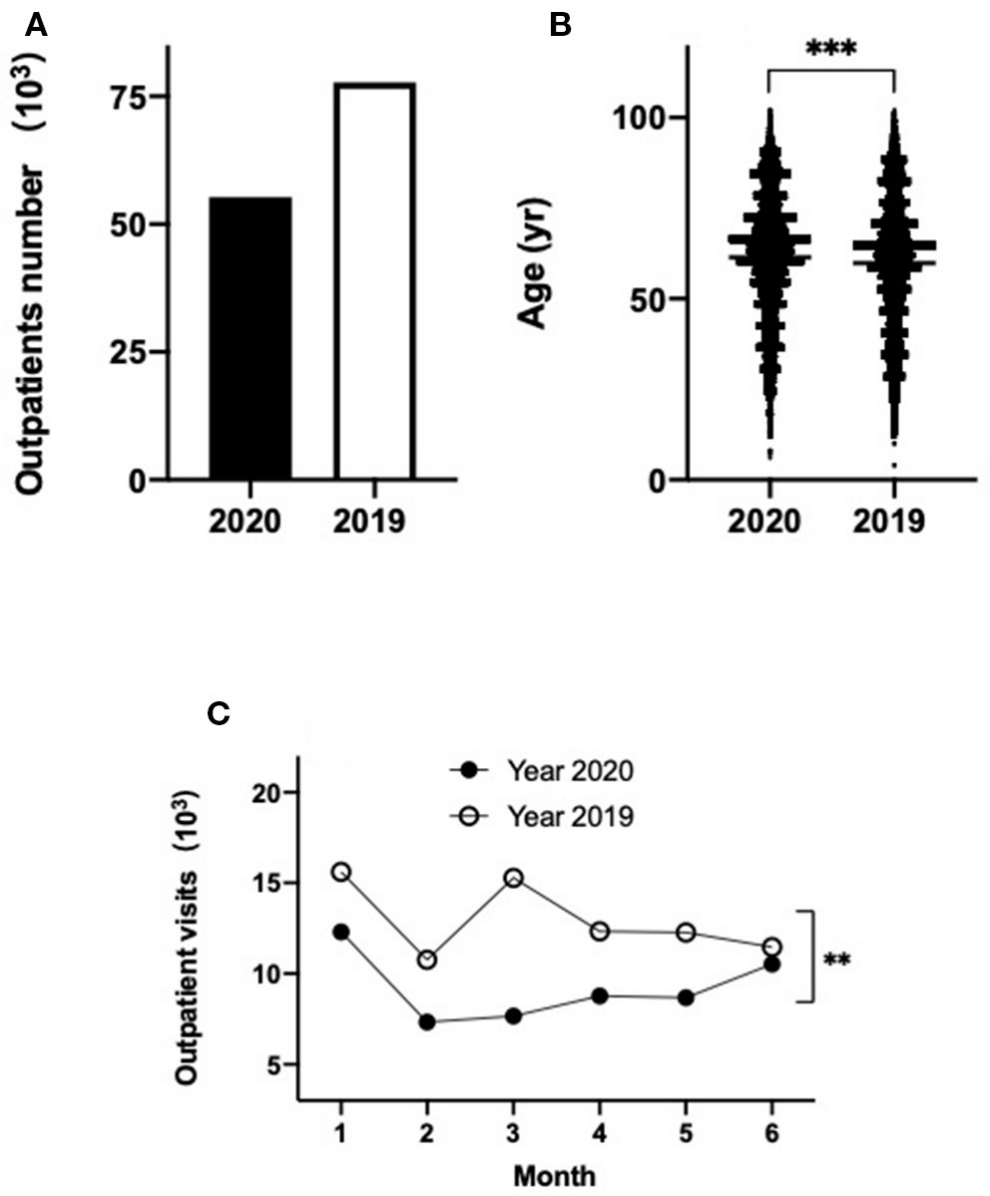
Outpatients visits at Tongren Hospital primary care clinics during the first 6 months of 2020 (black) compared to 2019 (empty) **(A)**; age distribution of patients in 2020 versus 2019 **(B)**; outpatients visits each month over the first six months of 2020 (black) and 2019 (empty) **(C)**. **p < 0.01, ***p < 0.001.

Table 1 summarizes the overall patient characteristics for this study. There was nearly a 30% drop in the total number of GP visits to the outpatients at Shanghai Tongren Hospital in the period from January to June in 2020, compared to the same period in 2019 (55,293 vs. 77,706) (Figure 1A). The average age of the patients was significantly higher over these 6 months in 2020 than that in 2019 (61.43 ± 0.1 vs. 59.89 ± 0.1 years; *p* < 0.001) (Figure 1B). In the first 6 months of 2020, the overall outpatient numbers were decreased by 21, 32, 50, 29, 29, or 8%, respectively, compared with the same period in 2019 (Figure 1C; *p* < 0.01), the biggest difference between these 2 years was observed in March. Additionally, a dip in visits was observed in February in both years, which corresponds with the Chinese New Year holiday period (January 24, 2020). The 2019 data show two peaks, one in January and one in March, which correspond to the peak of winter-associated illnesses and a rebound in demand following Chinese New Year, respectively. A rebound was not observed in March 2020.

**Table 1:** Demography of patient visits in the first 6 months of 2020 during the first wave of the COVID-19 outbreak, compared to corresponding data from 2019.

**Characteristics**	**Jan–Jun 2019**	**Jan–Jun 2020**	***P*-value**
**Total number**	77,706	55,293	–
**Age, mean (SEM), yrs**	59.89 (0.1)	61.43 (0.1)	<0.001
**Age groups**, ***n*** **(%)**			
<40 yrs	12,451 (16.0)	7,102 (12.8)	<0.001
40–64 yrs	32,683 (42.1)	23,117 (41.8)	0.36
≥65 yrs	32,572 (41.9)	25,074 (45.3)	<0.001
**Sex**, ***n*** **(%)**			
Male	34,096 (43.9)	24,768 (44.8)	<0.001
Female	43,610 (56.1)	30,525 (55.2)	<0.001
**Cost, mean (SEM) (¥)**	264.7 (1.0)	347.9 (1.5)	<0.001
**Diseases**, ***n*** **(%)**			
Cardiovascular	29,636 (38.1)	21,048 (38.1)	0.79
Respiratory	17,406 (22.4)	7,904 (14.3)	<0.001
Endocrine	9,072 (11.7)	7,034 (12.7)	<0.001
Gastrointestinal	9,261 (11.9)	7,000 (12.7)	<0.001
Neurology	3,884 (5.0)	3,629 (6.6)	<0.001
Urology	1,119 (1.4)	1,028 (1.9)	<0.001
Musculoskeletal	1,531 (2.0)	1,436 (2.6)	<0.001
Hematology	372 (0.5)	316 (0.6)	0.02
Others	5,425 (7.0)	5,898 (10.7)	<0.001
**Psychology**, ***n*** **(%)**			
Depression	163 (0.2)	74 (0.1)	<0.01
Anxiety	288 (0.4)	319 (0.6)	<0.001
Insomnia	1,099 (1.4)	1,403 (2.5)	<0.001
Total	1,550 (2.0)	1,796 (3.2)	<0.001

### Demographic Characteristics

**Figure 2:**
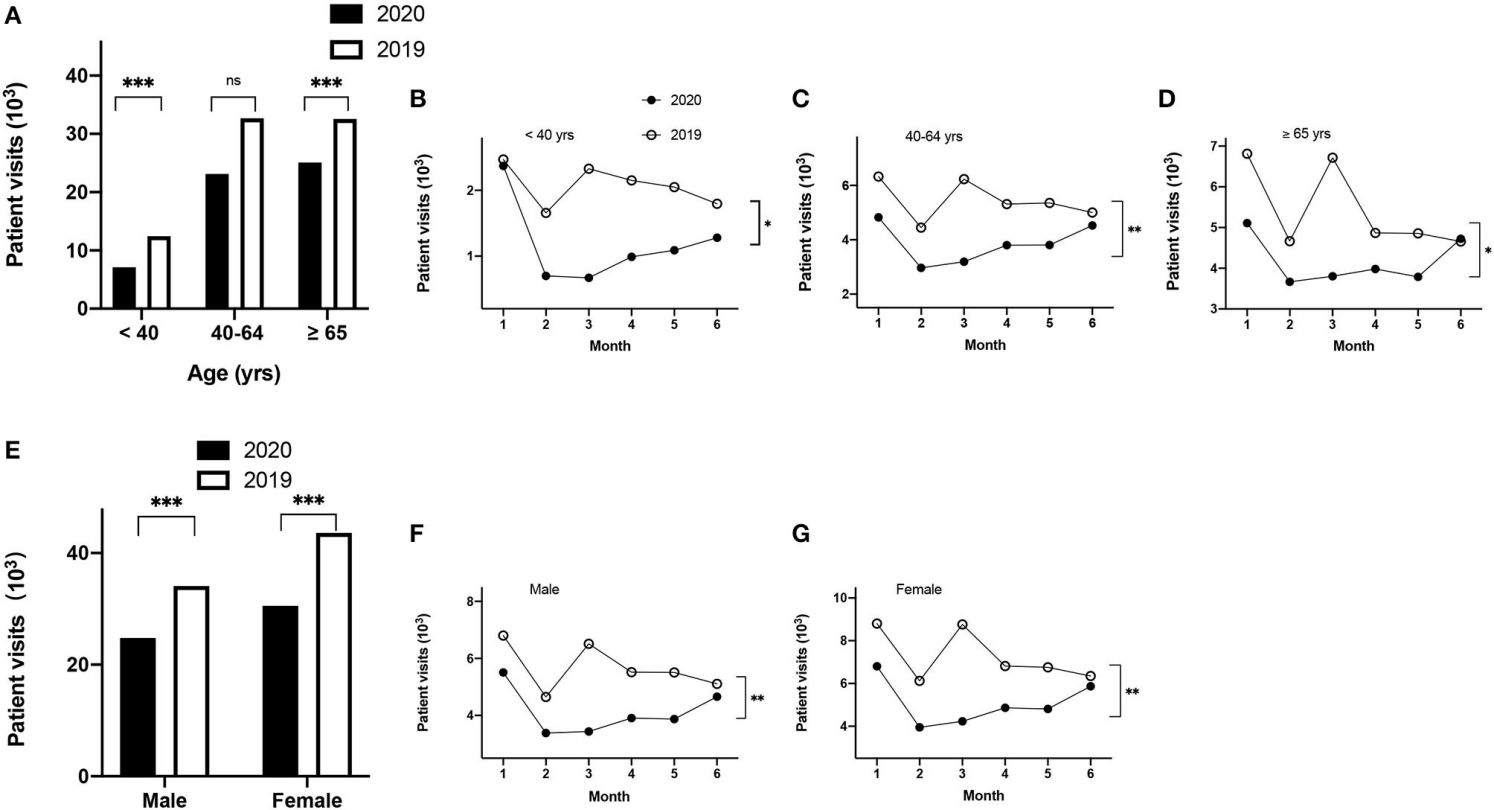
Demographics of patient visits stratified by age and sex. Patients visits for the three aged groups over the first 6 months (<40, 40–64, ≥65 years) **(A)**; monthly distribution of patients visits for the subgroups <40 years **(B)**; 40–64 years **(C)**, and ≥65 years **(D)**; patient visits for male or female patients in 2020 compared to 2019 **(E)**; monthly distribution of patients visits for males **(F)** and females **(G)**. *p < 0.05, **p < 0.01, ***p < 0.001.

To further determine if the age factor contributed to the change in outpatient visits during the COVID-19 period, these patients were divided into three groups: <40, 40–64, and ≥65 years. The number of outpatient visits decreased by 43, 29, or 23%, within the age groups of <40, 40–65, and ≥65 years, respectively, in 2020, compared to that in 2019 (Table 1, Figure 2A). However, only a significant difference between the 2020 and 2019 was observed for the age groups <40 or ≥65 years (*p* < 0.001), but not in the 40- to 65-year age group (Table 1, Figure 2A). When the outpatient visits were analyzed on a monthly basis, in the patients' group <40 years, there was no obvious difference between these 2 years within January (Table 2, Figure 2B). However, within the subsequent months, the visits by patients dropped by 58, 71, 54, 47, or 29% in February, March, April, May, and June in 2020, respectively, compared to the corresponding months in 2019 (Table 2, Figure 2B; *p* < 0.05). However, within the 40- 65-year group, the outpatient visit numbers dropped by 24, 33, 49, 28, 29, or 10% from January to June in 2020, compared to the outpatient visits during the same period of 2019 (Table 2, Figure 2C; *p* < 0.01). Finally, in ≥65-year group, the outpatient visits in the first 5 months dropped by 25, 21, 43, 18, or 22%, compared with the same period of 2019, but the outpatient visits returned to a similar level by June 2020 compared to June 2019 (Table 2, Figure 2D; *p* < 0.05). Interestingly, there was a significant difference between 2020 and 2019 among all these three groups when the monthly trend of outpatient visits was analyzed (Table 2, Figures 2B–D; *p* < 0.05). Notably, for all age groups, the largest fall in monthly visits during 2020, compared to 2019, occurred in March 2020.

**Table 2:** The monthly patients number from January to June in 2020 and 2019, with age, sex, disease in major systems and psychological disorders.

**Characteristics**	**2019**						**2020**						***P* value**
	**Jan**	**Feb**	**Mar**	**Apr**	**May**	**Jun**	**Jan**	**Feb**	**Mar**	**Apr**	**May**	**Jun**	
**Age groups**													
<40 yrs	2,469	1,658	2,328	2,151	2,050	1,795	2,372	700	672	989	1.089	1,280	0.01
40–64 yrs	6,328	4,450	6,232	5,314	5,357	5,003	4,828	2,965	3,194	3,800	3,805	4,525	<0.01
≥65 yrs	6,812	4,663	6,716	4,866	4,857	4,657	5,109	3,668	3,803	3,981	3,789	4,724	0.03
**Sex**													
Male	6,804	4,646	6,513	5,517	5,509	5,107	5,508	3,384	3,438	3,906	3,874	4,658	<0.01
Female	8,805	6,125	8,763	6,814	6,755	6,348	6,801	3,949	4,231	4,864	4,809	5,871	<0.01
**Diseases**													
Cardiovascular	5,905	3,957	5,930	4,781	4,646	4,417	4,425	3,236	3,138	3,464	3,058	3,727	<0.01
Respiratory	3,961	2,914	3,251	2,743	2,580	1,957	3,548	1,183	774	732	680	987	0.01
Endocrine	1,855	1,262	1,774	1,372	1,387	1,422	1,344	1,025	1,004	1,058	1,116	1,497	0.02
Gastrointestinal	1,627	1,173	1,871	1,468	1,558	1,564	1,259	633	1,048	1,324	1,270	1,466	0.03
Neurology	717	473	745	646	666	637	621	411	514	626	723	734	0.52
Urology	216	124	203	179	205	192	169	95	122	159	213	270	0.61
Musculoskeletal	314	197	313	236	215	256	220	140	236	274	256	310	0.62
Hematology	65	59	63	67	65	53	54	31	40	49	71	71	0.21
Others	949	612	1.126	839	942	957	679	579	793	1.084	1.296	1.467	0.64
**Psychology**													
Depression	38	29	31	22	24	19	9	6	9	16	16	18	<0.01
Anxiety	59	50	62	35	47	35	46	43	56	55	68	51	0.40
Insomnia	203	148	231	156	173	188	189	158	208	260	294	294	0.09
Total	300	227	324	213	244	242	244	207	273	331	378	363	0.25

Next, we investigated if there was a difference between groups stratified by sex. Among all patients, 30,525 (55.2%) or 43,610 (56.1%) were female in 2020 or 2019 (*p* < 0.001), respectively (Table 1, Figure 2E). Among male patients, visits dropped 19, 27, 47, 29, 30, and 9% in January, February, March, April, May, or June in 2020, respectively, compared to the corresponding months in 2019 (Table 2, Figure 2F; *p* < 0.01). Similarly, for female patients, the visits dropped 23, 36, 52, 29, 29, 8% in the first 6 months of 2020 compared to the same period of 2019 (Table 2, Figure 2G; *p* < 0.01).

### Cost per Visit for the Hospital

**Figure 3:**
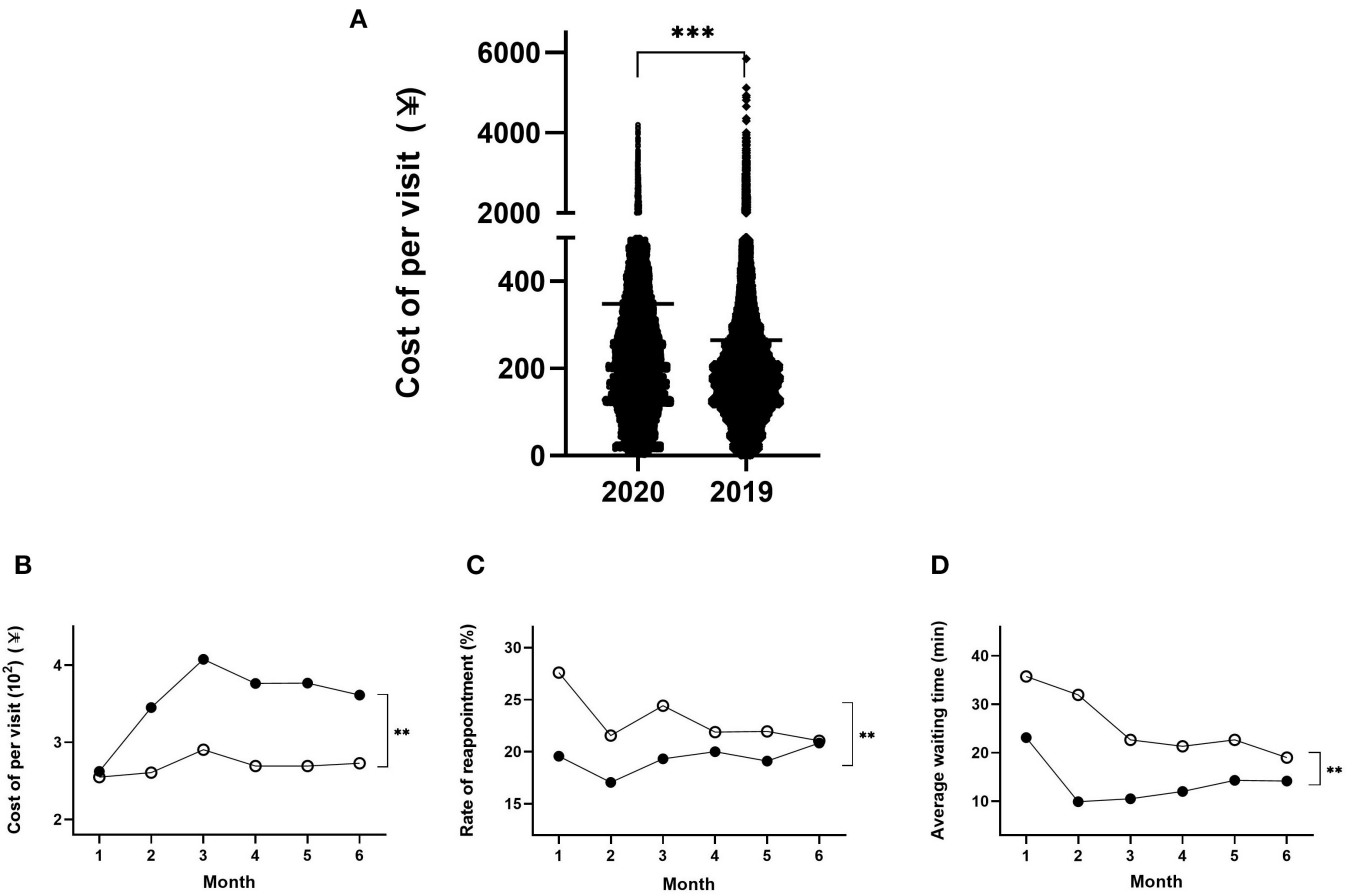
Demographics of the cost per visit among patients over the 6 months in 2020 and 2019 **(A)**; the monthly distribution of patients cost per visit **(B)**; reappointment rates **(C)** and average waiting time **(D)** in 2020 and 2019. **p < 0.01, ***p < 0.001.

The median cost of per patient visits to outpatients was 1.3-fold higher in the first half of 2020 compared to the same period in 2019 (¥347.9 ± 1.5 vs. 264.7 ± 1.0) (Table 1, Figure 3A; *p* < 0.001). The patients pay only a fraction of this cost, based on their income. When analyzed on a monthly basis, the cost per visit in January 2020 was similar to that of the same period in 2019 (Figure 3B). However, the cost per visit increased in February and peaked in March 2020, at a cost that was 1.55-fold higher than that in January 2020. The cost per visit decreased slightly, by ~10%, in April, May, and June 2020, compared to March 2020. Compared to the cost per visit each month in 2019, the cost per visit during February to June 2020 was increased by a similar amount over these 5 months, specifically, 1.32-, 1.40-, 1.40-, 1.40-, or 1.32-fold higher in 2020, compared to the corresponding months in 2019 (Figure 3B; *p* < 0.01).

By contrast, analysis of the rate of reappointment by month showed that the gap between the 2 years was largest in January and then progressively decreased between February and May, with almost no significant difference by June between these 2 years (Figure 3C; *p* < 0.01). Specifically, the rate of reappointment in the first 6 months of 2020 was decreased by 29, 21, 21, 9, 13, and 1% (*p* < 0.01), respectively, compared to the corresponding months in 2019.

Similarly, the average waiting time from January to June 2020 was significantly lower by 35, 69, 54, 44, 37, and 25% in each month, compared to the same periods during 2019 (Figure 3D; *p* < 0.01).

### Categories of Diseases

The patients' attendances were grouped into nine medical systems based on their most important medical diagnosis at each visit. These data are presented in Tables 1, 2.

**Figure 4:**
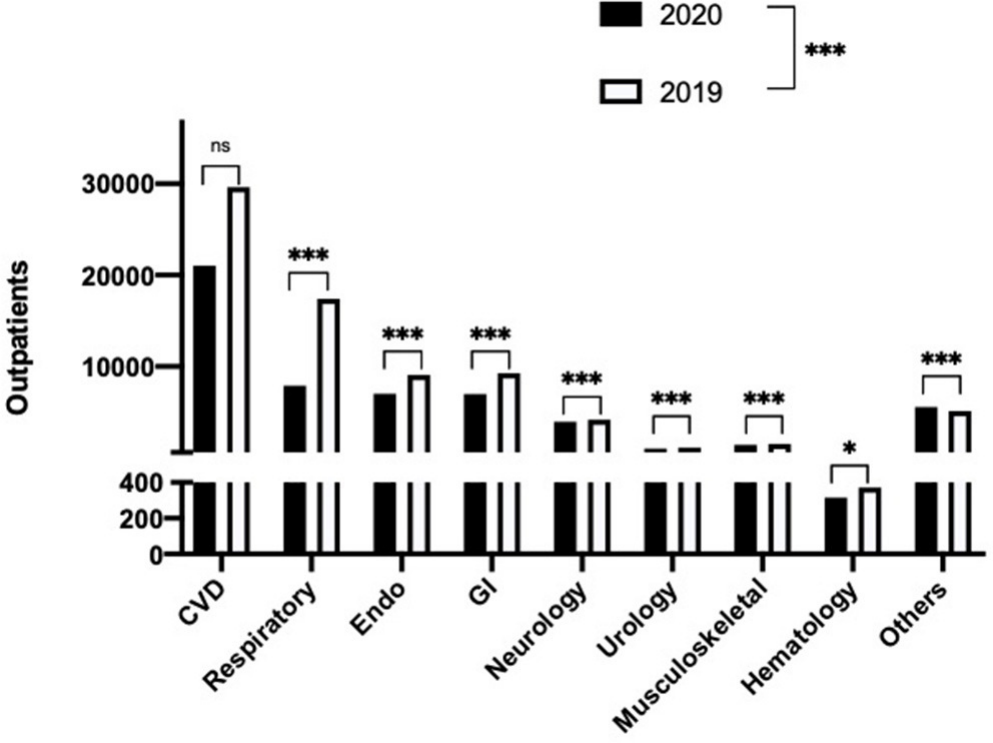
Subgroup analysis by primary diagnosis of patient visits for the eight major diagnostic systems, plus others (unclassified) in 2020 and 2019. *p < 0.05, ***p < 0.001.

The largest category of disease diagnosis was cardiovascular disease (CVD) (Table 1, Figure 4). The number of patients diagnosed with CVD was decreased by 29% in the first half of 2020 compared to the same period of 2019 (Table 1, Figure 4). Similar reductions in 2020, compared to 2019, were seen in other diagnostic categories of illness, specifically decreased numbers of patients with respiratory (55%), endocrine (22%), gastrointestinal (24%), neurological (7%), urological (8%), musculoskeletal (6%), or hematological (15%) diseases were observed (Figure 4). Interestingly, the number of patients with other diseases (except for the 8 systems described above) was increased by 9% in the first half of 2020 compared to that in 2019. Although the absolute number of the patients diagnosed with CVD decreased significantly, the proportion of these patients' number (not the absolute patients' number) was not significantly different between 2020 and 2019. However, a significant difference was observed for the proportion of all the other eight categories of illness between 2020 and 2019 (Table 1, Figure 4; *p* < 0.05).

**Figure 5:**
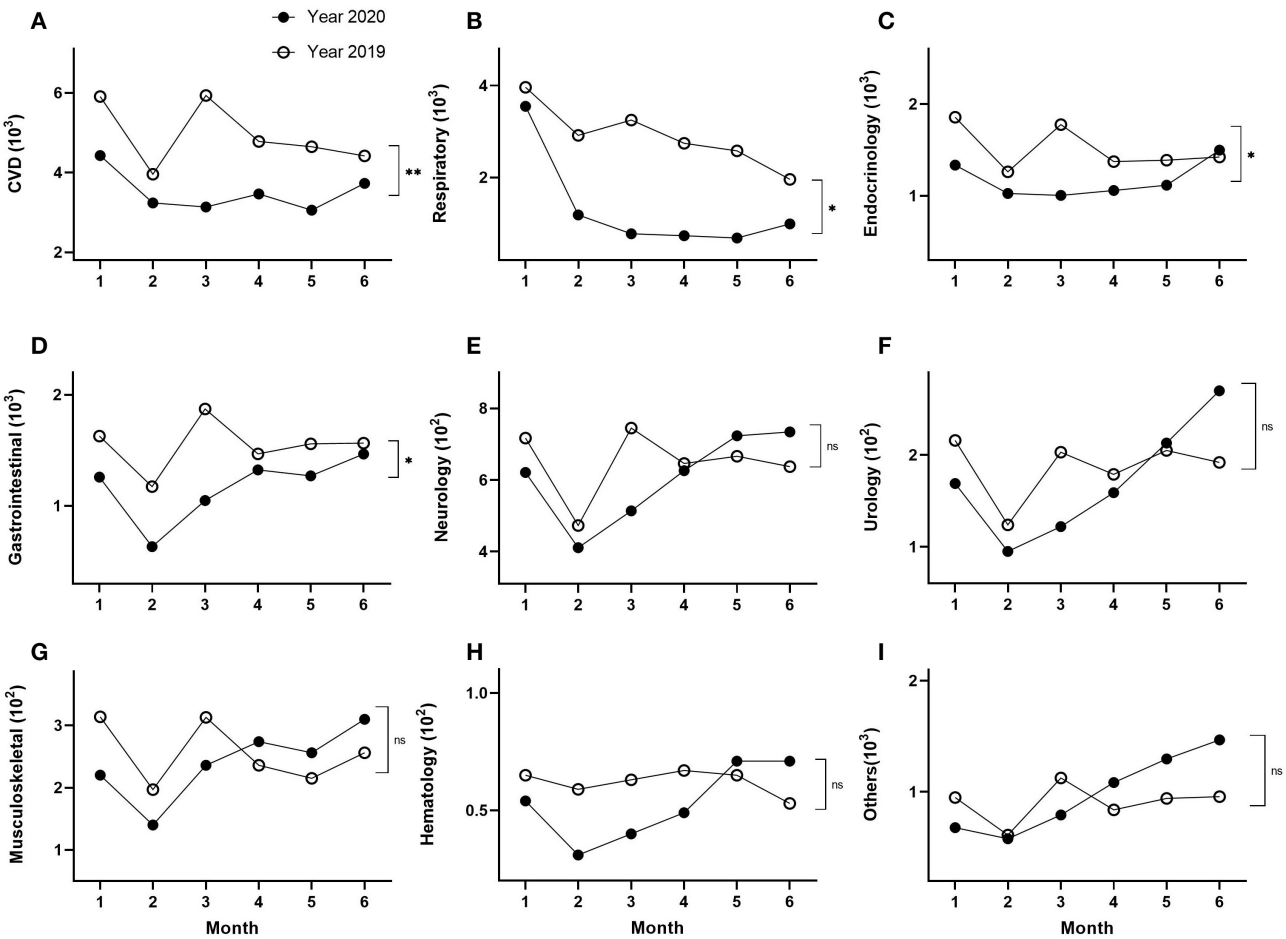
Subgroup analysis of monthly distributions of visits stratified by primary diagnosis, cardiovascular disease **(A)**, respiratory **(B)**, endocrine **(C)**, gastrointestinal **(D)**, neurology **(E)**, urology **(F)**, muscular-skeletal **(G)**, hematology **(H)**, and others (unclassified) **(I)**, respectively. *p < 0.05, **p < 0.01.

When examined by monthly trend, the number of patient consultations with CVD was decreased by 25, 18, 47, 28, 34, or 16% each month between January to June in 2020, compared to consultations over the same monthly periods during 2019 (Table 2, Figure 5A; *p* < 0.01). However, a more drastic drop for patient consultations with respiratory diseases was observed, showing a 10, 59, 76, 73, 74, or 50% drop from January to June in 2020, compared to the corresponding months in 2019 (Table 2, Figure 5B; *p* < 0.05). The patients with endocrine disease were decreased by 28, 19, 43, 23, or 20% between January and May, but then increased 5% in June 2020, compared with the corresponding months of 2019 (Table 2, Figure 5C; *p* < 0.05). The number of gastrointestinal disease patients was reduced by 22, 46, 44, 10, 18, or 6% each month from January to June during 2020, compared to the same period in 2019 (Figure 5D; Table 2; *p* < 0.05). Notably, the largest drop in consultations occurred in March 2020 for CVD (47%), respiratory disease (76%), endocrine disease (43%), neurological disease (31%), urological disease (40%), and other diseases (unspecified) (30%), whereas the largest drop in consultations occurred in February for gastrointestinal (46%) and hematological diseases (47%), and finally, the largest drop in consultation occurred in January for musculoskeletal disease (30%) (Table 2, Figures 5A–I).

Furthermore, with the exception of CVD, respiratory disease, and gastrointestinal diseases, a rebound of patient consultations was seen during the second half of the 6-month period under study, generally by April, May, or June 2020, where the number of consultations occurring in one or more of these months in 2020 exceeded the monthly consultations observed in the corresponding months in 2019. The diagnostic categories in which a rebound of cases was observed were endocrine (June), neurology (May), urology (May), musculoskeletal (April), hematology (May), or other diseases (April) (Table 2, Figures 5E–I).

A significant difference in the monthly trend data was observed among cardiovascular, respiratory, endocrinologic, and gastrointestinal diagnostic categories between 2020 and 2019 (Figures 5A–D; *p* < 0.05), but there was no significant difference of monthly trend among neurology, urology, musculoskeletal, hematology, and other diseases between 2019 and 2020 (Table 2, Figures 5E–I).

### Psychological Factors

**Figure 6:**
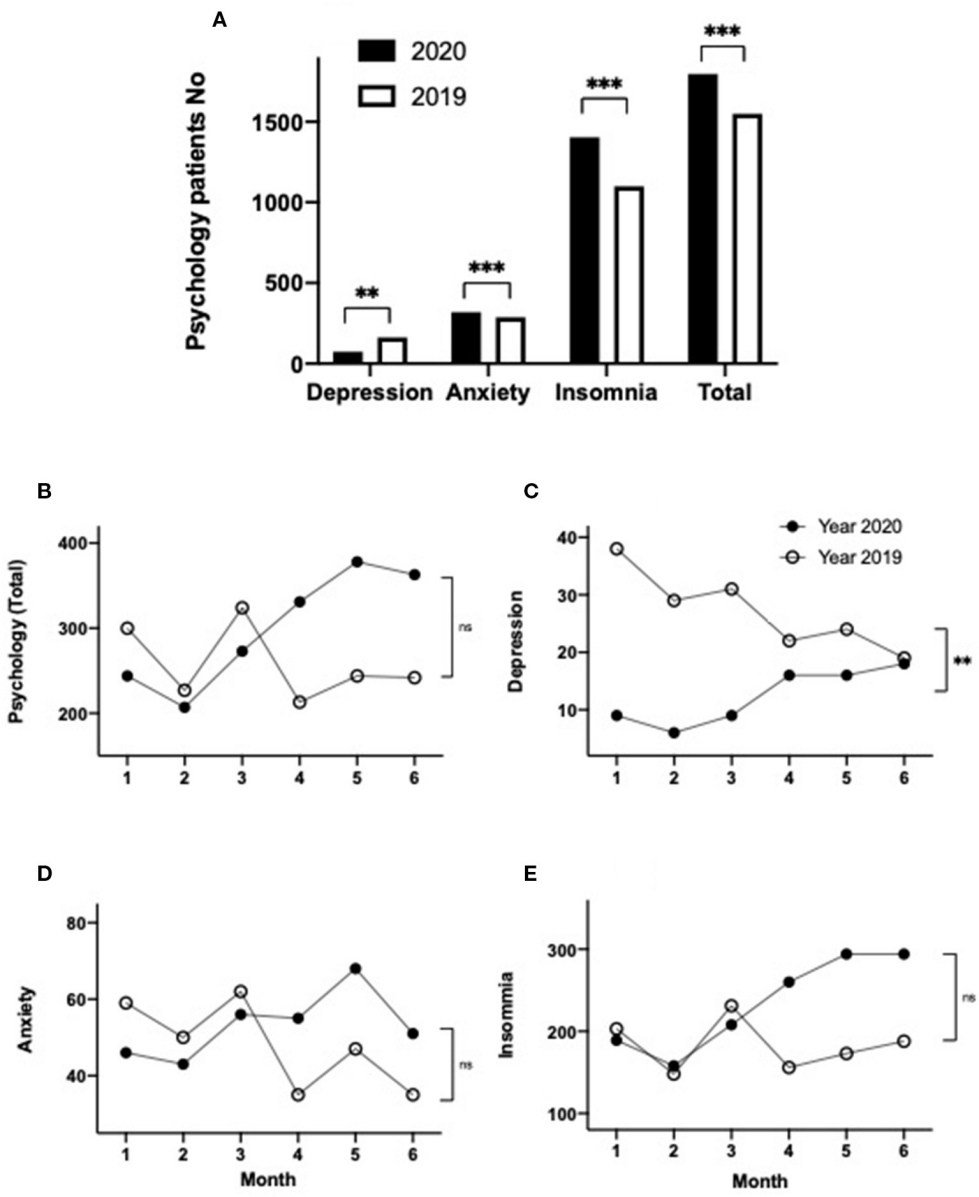
Demographics of patient visits with a primary diagnosis of psychological disorders, including depression, anxiety, insomnia, and the total number of psychological visits in 2020 and 2019 **(A)**; subgroup analysis of monthly distributions of all psychology patients **(B)**, depression **(C)**, anxiety **(D)**, insomnia **(E)** over the first 6 months in 2020 and 2019. **p < 0.01, ***p < 0.001.

Patients whose primary diagnosis was of psychological disease were a subset of the other diseases (unspecified) diagnostic category. It has been reported that psychological factors contribute to the choice of activities undertaken by people generally, including decisions concerning visiting doctors (28). The total numbers of patients presenting with psychological problems in 2019 and 2020 were 1,550 and 1,796, respectively, which correspond to an increase of 1.2-fold in the first half of 2020 compared to 2019 (Table 1, Figure 6A; *p* < 0.001). To further understand these data, we undertook subgroup analysis by classifying all of the patients with psychological problems into three categories: depression, anxiety, or insomnia. The number of depression patients was substantially decreased by 55% in the first half year of 2020 compared to 2019 (Table 1, Figure 6A; *p* < 0.01), whereas the number of anxiety or insomnia patients was increased by 11 or 28%, respectively, in the first half of 2020 compared with that in the same period of 2019 (Table 1, Figure 6A; *p* < 0.001).

The monthly trend data for the total number of patient visits with psychological problems were lower during January to March 2020, compared with 2019 (Table 2, Figure 6B). However, during April to June 2020, the number of visits for psychological problems substantially exceeded the consultation numbers seen during the corresponding months of 2019 (Table 2, Figure 6B). When the monthly trend data for psychological illnesses were analyzed for each of the three psychological illness categories, substantial differences were observed. In the case of the patients with depression, the number of consultations was dramatically lower in the first 3 months of 2020 (a decrease of 76, 79, or 71% for January, February, and March 2020, respectively), compared to the corresponding months of 2019, while during the second 3 months of the study period, case consultations for depression trended back toward the corresponding 2019 levels (decreases of 27, 33, and 0.1% for April, May, and June 2020, respectively) (Table 2, Figure 6C; *p* < 0.01). By comparison, consultation numbers for anxiety or insomnia patients were similar or slightly below consultation levels seen in 2019 during January to March, whereas the number of consultations for anxiety and insomnia were substantially larger during April, May, and June 2020, compared to the corresponding months during 2019 (Table 2, Figures 6D,E). However, a significant difference between the 2020 and 2019 data was observed only for the depression group (*p* < 0.01), but not in the anxiety or insomnia groups or the total psychological group (Table 2, Figures 6B,D,E; *p* > 0.05).

### Correlation Between the Monthly Patients' Number vs. Reappointment, Average Waiting Time, Cost per Visit, or COVID-19 Confirmed Cases

**Figure 7:**
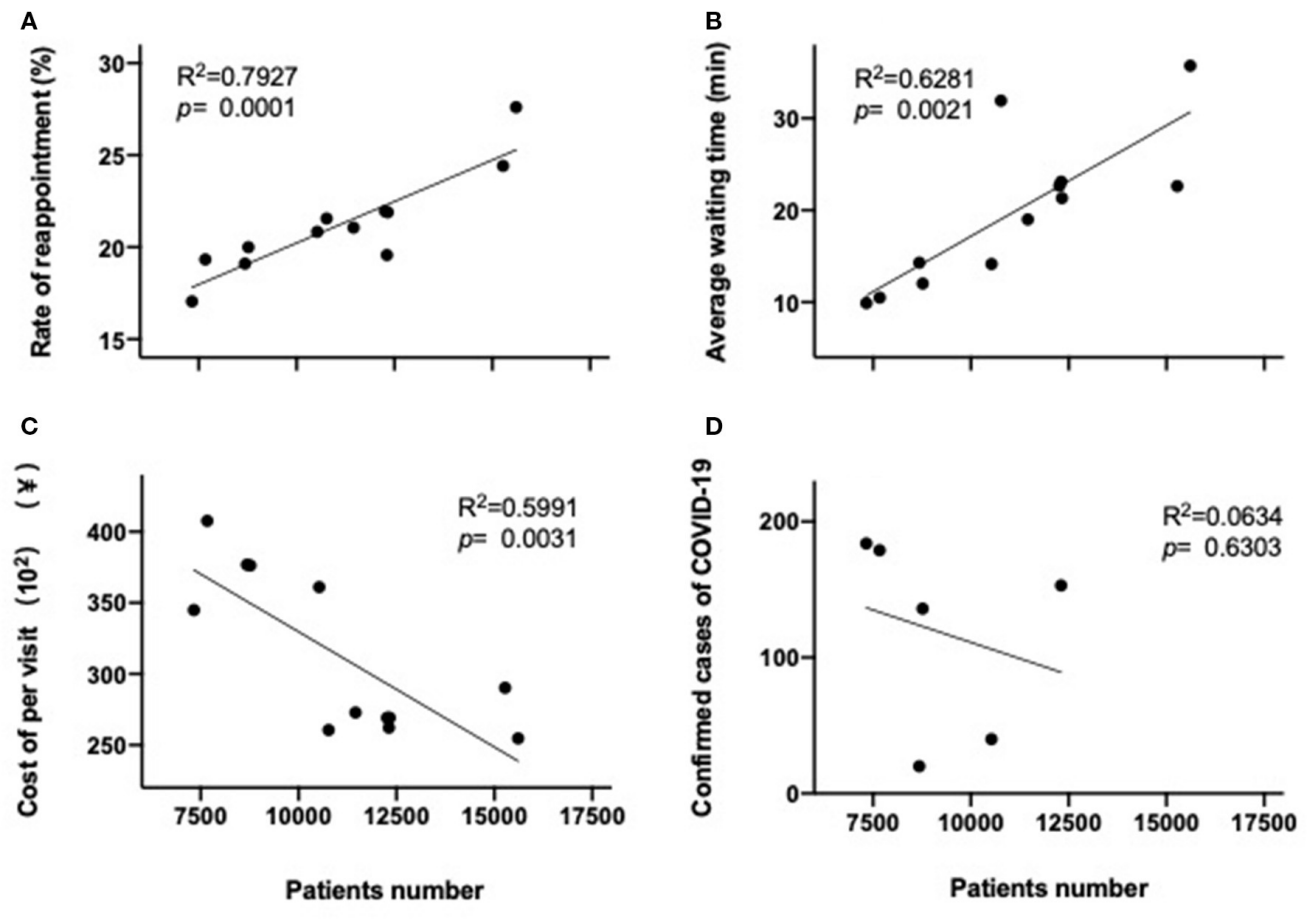
Correlation between patient visits per month and reappointment rate **(A)**, average waiting time **(B)**, or cost per visit **(C)** in the first 6 months of both 2020 and 2019, or number of confirmed COVID-19 patients in Shanghai **(D)** in the first 6 months of 2020.

The number of patients who attended the outpatient clinics each month was determined for each monthly interval during the first 6 months of 2019 and 2020 (corresponding to a total of 12 data intervals). The rate of reappointment and average waiting time for each monthly interval were then determined, and these data were found to positively correlate with the number of patient visits for that month, for the combined data derived from the first half of both 2019 and 2020 (*R*^2^ = 0.79, *p* = 0.0001; *R*^2^ = 0.63, *p* = 0.002) (Figures 7A,B). By cost, the cost per visit was inversely correlated with the number of patients per monthly interval (*R*^2^ = 0.5991, *p* = 0.003) (Figure 7C). No significant correlation was observed between the outpatient visit number per monthly interval for the first 6 months of 2020 and the confirmed COVID-19 cases number for all of Shanghai during the same period in the first half of 2020 (Figure 7D).

### Lockdown

**Figure 8:**
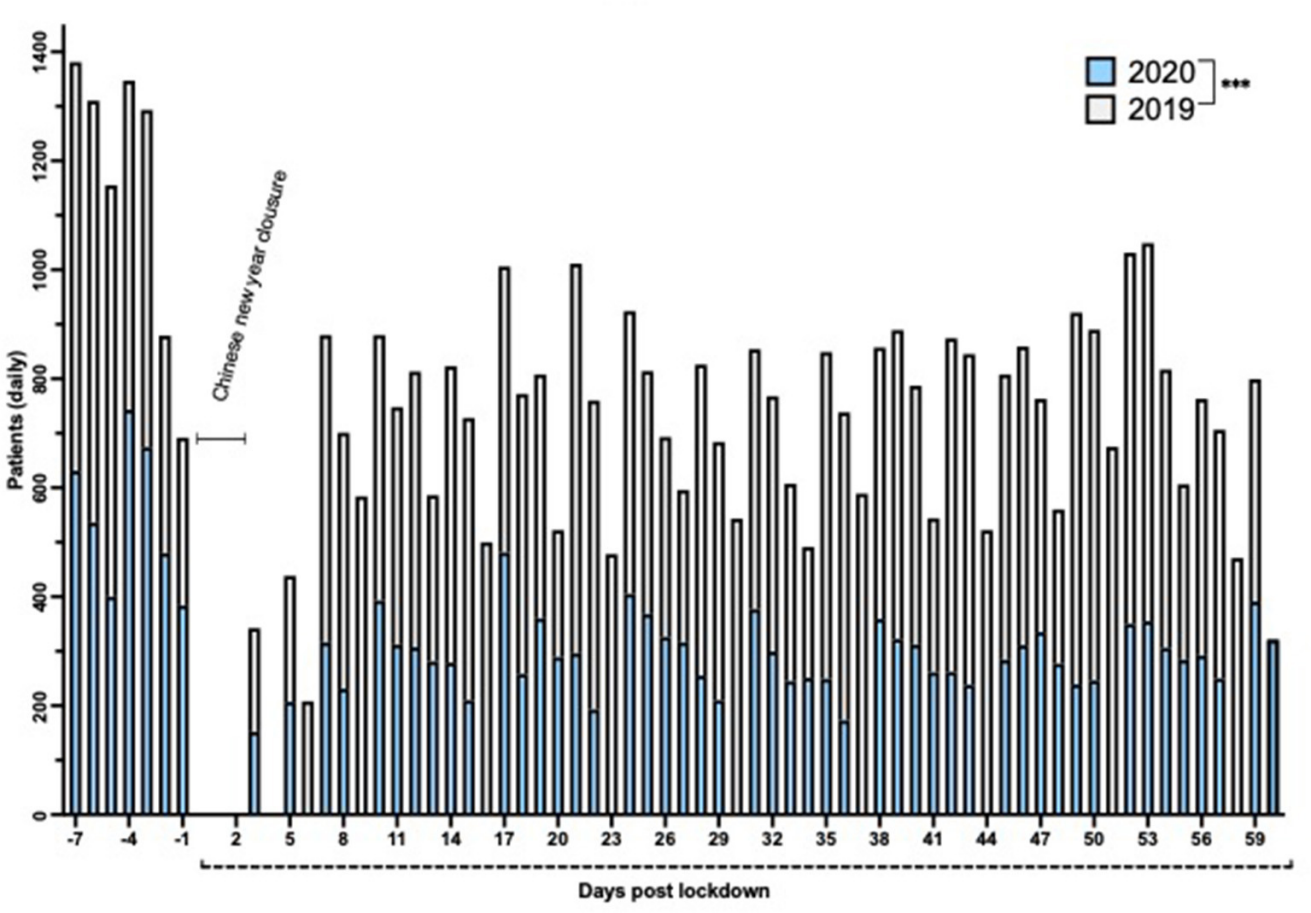
Number of daily patient visits over a 67-day period in 2020 (empty) compared to 2019 (gray). The days were aligned by Chinese New Year (February 5, 2019, and January 25, 2020). Day 1 corresponds to the first day of lockdown (January 24, 2020). ***p < 0.001.

During the period of lockdown in Shanghai (January 24 to March 24, 2020), the overall total number of outpatient visits was decreased to 40% compared with the same period of 2019 (Figure 8; *p* < 0.001). Figure 8 shows the daily number of patient visits for the period 7 days before lockdown (January 17, 2020), until 60 days after lockdown commenced during 2020. These 2020 data have been compared to the corresponding daily data from 2019, with the two data sets being aligned at the first day of Chinese New Year (February 5, 2019, and January 25, 2020). The rationale for this alignment is 2-fold: first, the Chinese New Year holiday substantially affects the pattern of consultations before, during, and after the holiday, and second, the commencement of lockdown occurred on New Year's Eve (January 24, 2020). Several features emerge from Figure 8. First, from day 10 until day 60 after lockdown, a 7-day periodicity can be observed during both 2019 and 2020, corresponding to the normal working week. Second, zero patient attendances are observed for the 3 days of clinic closure corresponding to the Chinese New Year closure during 2019 (February 5–7, 2019) and 2020 (January 25–27, 2020), respectively. Third, a substantial reduction in patient visits occurred on all days contained within this graph, both during the week before lockdown/Chinese New Year and during the 10 weeks after lockdown.

**Figure 9:**
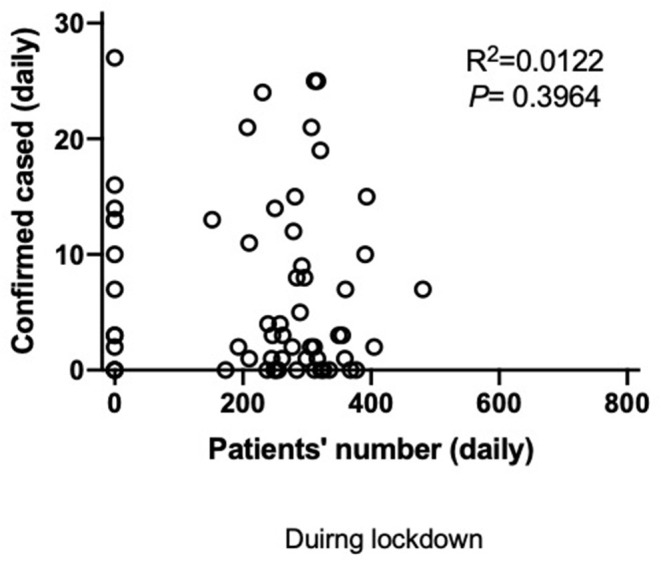
Correlation between the number of daily visit to outpatients in Tongren Hospital and the number of confirmed COVID-19 patients within Shanghai.

Finally, when analyzed on a daily basis, there was no significant correlation between the outpatients' visits each day and the number of confirmed COVID-19 cases on that day (*R*^2^ = 0.0122, *p* = 0.3964) (Figure 9) during the lockdown period in Shanghai.

## Discussion

Our current study was a simple cross-sectional analysis without adjustment for any confounders or secular changes during the 18-month period of the study. In the current study, we have demonstrated that outpatient visits in primary care dropped 30% in the first half of 2020, compared with the same period of 2019. We believe this observation is most likely due to the pandemic of COVID-19 over this 6-month interval for the following reasons. First, the health authorities actively discouraged patients from engaging in face-to-face visits to hospital/outpatient departments unless it was absolutely essential but instead encouraged telephone and/or online consultations (29, 30). Second, we hypothesize that the patients were fearful of possible transmission of COVID-19 while moving through the community and especially attending a health care facility, such as the outpatient clinic, because media reports correctly emphasized a greater chance of being contaminated in these high-disease areas (2), influencing their decision to limit attendance at outpatient clinics.

Additionally, a restriction order from the authorities was made to limited social activities (31), especially during the lockdown period in Shanghai (32), which emphasized that hospital visits should be curtailed unless medical review became absolutely necessary. A drastic drop in Outpatient visits was observed in the week before lockdown (January 17–24, 2020) and in February 2020, which was mainly due to the strong message/instruction from the health authority that advised people to minimizing exposure within their environment, especially within hospitals (33). In addition, a reduction in the outpatient visits was observed during the traditional Chinese New Year (January 24–February 2), during which there is a closure of outpatient clinics for 3 days. Notably, Chinese culture also supports an avoidance of seeing doctors within the New Year period (34). These factors are all consistent with the reduced outpatient visits observed during late January and February 2020.

When attendance at outpatients was stratified by age, the largest fall in attendances was observed among relatively younger patients (<40 years), who may have less critical medical conditions, and/or may be able to cope better for a longer period of time while staying at home without consulting a doctor, whereas the elderly patients are more likely to have more severe preexisting medical conditions and therefore may have to seek medical attention more quickly (35). Additionally, while all patients were encouraged to use telephone or online consultations where possible by the health authorities, to minimize potential exposure to the COVID-19 outbreak (36), younger patients may be more likely to engage with technology-driven consultations, such as video consultations, compared to older patients (37).

On the other hand, among the senior age group (≥65 years), the observed reduction in outpatient visits might also be due to a combination of both the restriction orders from the authorities, as stated above, and persistent media reporting, where there was a strong message correctly stating that elderly patients are more vulnerable to SARS-CoV-2 viral attack (38), which is likely to have also contributed to limiting the inclination of elderly patients to attend hospitals/outpatient clinics, in addition to their generally reduced mobility (39).

Finally, within the middle-aged group (40–64 years), it was not surprising that there was no significant difference in the outpatient visits in this group, because these patients are more mobile, and they likely perceived that their risk of contracting/dying from this potential viral attack was minimal. This perception was supported by extensive media reporting that risk of death was primarily increased by the presence of preexisting chronic conditions, which are relatively few within this middle-aged group.

Despite restrictions beginning to ease by April 2020 (restrictions were eased on March 24, 2020), it is surprising that the outpatient visits were persistently low in the following months, that is, April and May 2020. By contrast, the outpatient visits in March, April, and May 2019 returned to similar levels observed during January 2019, with a dip in consultations only occurring in relation to Chinese New Year in February 2019. Finally, outpatient visits returned to similar levels in June 2020, comparing 2020 and 2019, which probably is due to the population gaining confidence that effective control of COVID-19 had been achieved, following the restrictions implemented against SARS-CoV-2 during the first 5 months of 2020 (40).

When outpatient visits data were stratified by sex, a reduction in both male and female patients was observed during the first half of 2020, compared to that in 2019. Of interest, the proportion of male patient visits was slightly but significantly higher in 2020 compared to that in 2019. Although there is a minor difference between male and female patients, the significance is negligible and will not be discussed further.

The outpatient visits monthly trend data demonstrated that outpatient visits were decreased by ~20% during January 2020, compared to visits in 2019, which was probably largely due to the timing of the Chinese New Year (January 25, 2020), while the Chinese New Year occurred a week later in 2019 (February 5, 2019). Nevertheless, attendances specifically during the week prior to Chinese New Year/lockdown in 2020 had fallen substantially, when compared to the week before Chinese New Year in 2019, indicating that a heightened awareness of the risk of COVID-19 may have already occurred among the community in Shanghai. Subsequently, the extent of the fall in outpatient visits continued to become and remained lower during February to May for each month in 2020, compared to that in 2019, which was probably due to the direct impact of COVID-19 (41), for the reasons stated above. However, patient visits returned almost back to the same levels in June 2020 compared to 2019, which we believe is due to the outcomes of effectively controlling COVID-19, which reduced anxiety concerning the pandemic (42). There was no difference in the pattern(s) of monthly distributions of attendances in male or female patients within the first half year 2020, compared to that of 2019. The similar distributions observed were not too surprising, for the reasons explained above.

We also observed that the average cost per visit in the first half year in 2020 was significantly higher than that in 2019, especially during February and March 2020. We believe that the higher cost was due to several potential factors. First, patients preferred and were encouraged to minimize the number of visits that they needed to make to the clinics and so chose to accumulate and discuss the health problems they may have in one consultation, rather than several consultations. Similarly, patients and their doctors chose to prescribe larger quantities of medications and other health interventions per visit, to compensate for the longer gap between visits, an approach that was encouraged by the Shanghai Health Authority for minimizing cross contamination, especially for COVID-19 (43). Monthly distribution of costs reached a higher plateau during April, May, and June 2020, compared to the same period during 2019, possibly due to the changed habits that had developed during the COVID-19 outbreak.

Both patient's concerns and the health policies promulgated by the health authorities may also explain the substantially reduced rate of reappointment in the first half year of 2020, compared to that in 2019. The reduced waiting time may be a consequence of both a reduction in the overall number of outpatients and as a consequence of health authorities attempting to minimize the time spent within health facilities, to minimize the risk of transmission. These data are also consistent with the data concerning decreased outpatient visits and increased costs described above.

Patient attendance data were also evaluated by the disease diagnosis category. The number of CVD patient attendances was decreased by almost 30% in the first half year of 2020, which is consistent with the overall reduction in attendances during COVID-19, for the general reasons discussed above (44). However, notably this reduction persisted throughout the period to June 2020, suggesting that patients may also have perceived, correctly, that chronic CVD placed them at additional risk of more severe COVID-19 disease. Consequently, these data support the possibility that patients with CVD were deliberately avoiding attending outpatients, because of their well-founded anxiety about their specific risk due to cardiovascular morbidity. These patient perceptions were likely further enhanced by the extensive media coverage that emphasized the increased risk of COVID-19 for patients suffering from chronic CVD. This media coverage is likely to have received additional traction with the public because of the slightly simplistic focus within the media that emphasized that the heart and major organs express a high level of ACE2, which is the binding site for COVID-19 (45). Notably, the role of ACE2 in severe morbidity and mortality during COVID-19 infection is substantially more complex (45).

Similarly, there was a >50% reduction in respiratory disease patient attendances. The possible explanations for these data encompass both media reporting, which emphasized that the first organ to be attacked by SARS-CoV2 and the most likely organ to fatally fail is the lungs (2), combined with more general patient anxiety. An additional factor that may have contributed to reduced respiratory consultations is that restrictions, including wearing masks and social distancing, may have reduced the rate of other respiratory infections, such as the common cold and influenza, which would normally be increased during the winter months in China (46). Unfortunately, our data do not contain sufficient detail to evaluate this possibility in the context of the Tongren Hospital outpatient clinics. In addition, there were only 712 COVID-19 patients (12) among 25 million population (4) in Shanghai, supporting the substantially reduced respiratory consultations.

Endocrine, gastrointestinal, and hematology attendances dropped by ~20% during the first half of 2020, while there was a minimal fall in neurology, urology, and musculoskeletal attendances. It is reasonable to speculate that endocrine, gastrointestinal, and hematological disease symptoms may be able to be tolerated and deferred for a moderate period of time and were consequently seen by the population as being symptoms that were not critical for simply surviving. On the other hand, the diagnoses encompassed within neurological disease (e.g., stroke), urology (e.g., urinary retention), and musculoskeletal disease (e.g., painful and mobility-limiting arthritis; fractures) are all diagnoses that are more difficult or impossible to defer. Another interesting observation was that other (unclassified) diseases were increased, which might reflect anxiety and uncertainty among patients and a strong desire for reassurance and clarification as soon as possible.

A particularly important set of observations that emerges from our data relates to psychological illnesses. Our results found a higher number of outpatient visits for overall psychological-related issues in the first 6 months of 2020 compared to 2019, including, specifically, a rise in anxiety and insomnia. This rise was most pronounced during April to June, which was after community transmission of COVID-19 infection had largely been overcome and the lockdown had been eased, which may be due to the chronicity of the psychological impact of COVID-19 on the general population, resulting in a quite long-term and heavy residual psychological impact (47). The rise in anxiety and insomnia is understandable, as these conditions are often related to acute psychological stress (48).

However, disturbingly, we observed that consultations for depression fell during the first half of 2020, compared to that in 2019 and only returned to normal levels in June 2020. Depression is a relatively more severe condition than anxiety and insomnia (49) and is characterized by a lack of overall motivation, which may include an increased reluctance to consult with health care professionals. A combination of media advice and lockdown rules may have compounded the lack of motivation among depressed patients, leading to a potential serious deficit in their care. Depression is known to be worse in winter, sometimes referred to as seasonal affective disorder (50), so the level of consultation for depression during January 2019 was higher than during June 2019. However, the monthly data for outpatients attendances during 2020 show a profound reduction (>80%) in January 2020, with a subsequent flattening of consultations for depression during the first half of 2020. The immediacy of the reduction in consultations for depression in January 2020 may reflect the highly sensitive nature of depression, where a relatively small trigger to decrease motivation may have a profound impact on seeking help. Overall, as COVID-19 was controlled and people were releasing from lockdown from late in March 2020, people's confidence was built up, and activities, including hospital visits, started to return toward normal (51).

There was a significant correlation between the monthly patients number and reappointment or waiting period for each month during the first half year of 2020, which is consistent with the gradual increase in patient numbers occurring as the COVID-19 crisis settled, and there was a build-up in confidence (52). There was an inverse correlation between cost per visit and patient numbers in the current study. This may be due to more frequent visits during the latter part of the first 6 months of 2020, requiring less/smaller prescriptions (43). However, no correlation was observed between the number of COVID-19 cases diagnosed and patient numbers attending outpatients, which may be due to the very small numbers of COVID-19 cases in the city (712 only in Shanghai among a population of 25 million). A larger study that incorporates data from the whole country may be able to demonstrate such a correlation. Overall, these data that demonstrate a reduction in COVID-19 infections to near zero by the end of lockdown further support the successful nature of the restriction orders utilized in controlling transmission of COVID-19 in Shanghai (53).

We acknowledge that there are limitations in the current study. First, this was a single-center study, although the hospital covers >700,000 members of the general population. In the future, we will conduct a multiple-center study with a larger pool size and different ethnic backgrounds. Second, there were no data from the telephone and/or online consultations included in the current study, which might partly explain the reduced numbers of face-to-face clinic consultations. Third, there were no COVID-19 patients included, as these patients were managed by the specialized infectious diseases wards in certain hospitals only, in addition to which there were very few COVID-19 patients within this 6-month period in Shanghai.

In conclusion, our data demonstrate some of the key parameters that were affected by the impact of COVID-19 on GPs and patients within Shanghai. Substantially reduced outpatient visits, especially among respiratory patients, were observed, probably due to general social distancing and the wearing of face masks. Of particular importance was our observations concerning the acute psychological impact on the general population and the need for GPs to be aware of this issue and proactively develop strategies to reduce associated morbidity in this area, particularly among those with a preexisting disorder, such as depression, during the outbreak of COVID-19, and in the future during similar pandemics. Telephone or online consultation may be an effective alternative approach during the outbreak of COVID-19 and future pandemics.

## Data Availability Statement

The raw data supporting the conclusions of this article will be made available by the authors, without undue reservation.

## Ethics Statement

The studies involving human participants were reviewed and approved by the Medical Ethics Committee of the hospital (No. 2020-079-01). The patients/participants provided their written informed consent to participate in this study.

## Author Contributions

ZX designed the experiments, collected data, performed and analyzed the experiment, and wrote the manuscript. JF designed the experiments, analyzed data, and wrote the manuscript. JD, XF, ST, and LQ collected and analyzed the data. KT and BH designed the experiments and revised the manuscript. SB designed the experiments and revised the manuscript. All authors contributed to the article and approved the submitted version.

## Conflict of Interest

The authors declare that the research was conducted in the absence of any commercial or financial relationships that could be construed as a potential conflict of interest.
